# Associations between triglyceride-glucose-derived indices combined with the atherogenic index of plasma and the precise severity of newly diagnosed coronary artery disease: a quantile regressions analysis

**DOI:** 10.3389/fnut.2026.1929245

**Published:** 2026-08-28

**Authors:** Runzhe Wu, Congying Wang, Lulu Pan, Jia Zhu, Haodong Jiang, Yongquan Niu, Feiyu Chen, Mengting Liu, Siqi Deng, Yunpeng Jin

**Affiliations:** 1 Department of Cardiology, The Fourth Affiliated Hospital of School of Medicine, and International School of Medicine, International Institutes of Medicine, Zhejiang University, Yiwu, China; 2 Department of Biostatistics, Key Laboratory of Public Health Safety of Ministry of Education, NHC Key Laboratory for Health Technology Assessment, School of Public Health, Fudan University, Shanghai, China

**Keywords:** atherogenic index of plasma, composite biomarker indices, coronary artery disease, C-reactive protein-triglyceride-glucose index, Gensini score, quantile regression approach, triglyceride-glucose index

## Abstract

**Background:**

Metabolic dysfunction, including insulin resistance, inflammation, and dyslipidaemia, plays a critical role in the development of coronary artery disease (CAD). The triglyceride-glucose (TyG) index, the C-reactive protein-triglyceride-glucose index (CTI), and the atherogenic index of plasma (AIP) are accessible biomarkers reflecting these disturbances. However, conventional methods of mean regression result in a loss of detailed information on disease severity. This study is aimed at evaluating the associations of the TyG index, CTI, and AIP with the quantitative severity of CAD across its entire distribution using quantile regression analysis.

**Methods:**

This retrospective study included 2,885 patients with newly diagnosed CAD, as confirmed by coronary angiography. CAD severity was quantitatively assessed using the Gensini score. Metabolic indices, including the TyG index, CTI, and AIP, were calculated, and their composite indices—the TyG-AIP and CTI-AIP—were also derived. Correlation analysis and quantile regression analyses were performed to evaluate the associations between these indices and CAD severity across the distribution of the Gensini score.

**Results:**

All the metabolic indices were positively but weakly linearly correlated with the Gensini score (all *p* < 0.001). The CTI demonstrated the strongest linear correlation with the Gensini score (partial *r* ≈ 0.29, adjusted for multiple covariates). Quantile regression analyses revealed significant heterogeneity in the associations between metabolic indices and CAD burden across the distribution of Gensini scores. All the metabolic indices were positively correlated with the Gensini score (all *p* < 0.001), with the CTI showing the strongest adjusted linear correlation. Quantile regression demonstrated progressively stronger associations across higher conditional quantiles, which remained significant after multivariable adjustment. Compared with the individual indices, the CTI-AIP and TyG-AIP showed stronger associations across most quantiles. Equality-of-slope testing confirmed significant heterogeneity across quantiles for all indices (all *p* < 0.01).

**Conclusion:**

TyG-derived indices and AIP were significantly associated with Gensini scores. Quantile regression revealed marked heterogeneity in these associations, with CTI-AIP showing the strongest effect after multivariable adjustment in patients with moderate to high Gensini scores. Clinically, these indices may serve as potential biomarkers, with significant association across the distribution of coronary heart disease burden, especially in patients with newly diagnosed CAD.

## Graphical abstract

**Figure:**
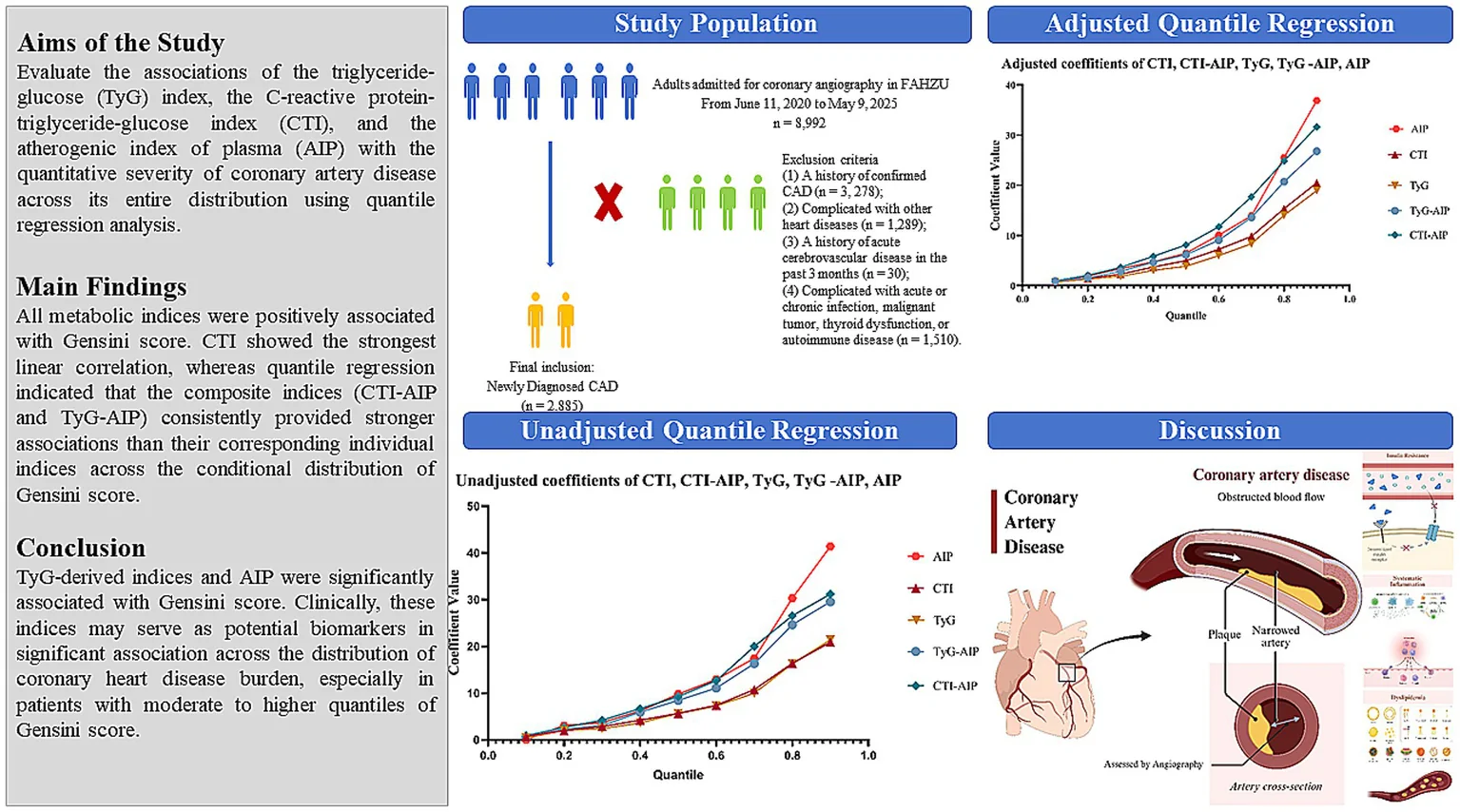
Created in BioRender. WU, R. (2026) https://BioRender.com/12l3q7a.

## Highlights

What is currently known about this topic?

1. Metabolic dysfunction, including insulin resistance, inflammation, and dyslipidaemia, plays a critical role in the development of coronary artery disease (CAD).
2. The triglyceride-glucose (TyG) index, the C-reactive protein-triglyceride-glucose index (CTI), and the atherogenic index of plasma (AIP) are accessible biomarkers that reflect these disturbances, and they are significantly associated with CAD.

What is the key research question?

How can the quantile heterogeneity between metabolic indices and the quantitative severity of CAD be explored?

What is new?

1. TyG-derived indices and AIP were significantly associated with Gensini scores.
2. Quantile regression revealed marked heterogeneity in these associations, with CTI-AIP showing the strongest effect after multivariable adjustment in patients with moderate to high Gensini scores.

How might this study influence clinical practice?

These indices may serve as potential biomarkers, with significant association across the distribution of coronary heart disease burden, especially in patients with newly diagnosed CAD.

## 1 Introduction

Coronary artery disease (CAD) is characterized by the atherosclerotic narrowing of the coronary arteries caused by the accumulation of atherosclerotic plaques, a condition that can result in a clinically silent or asymptomatic state. The accumulation of atherosclerotic plaques and the resulting luminal narrowing constitute the fundamental pathological mechanism responsible for this disease spectrum. Despite significant progress in understanding the disease pathophysiology and translational research efforts, the mortality rate of CAD continues to increase in China, the U. S., and many other countries, especially during and after the coronavirus disease 2019 (COVID-19) pandemic (1). In 2021, cardiovascular disease was the leading cause of mortality among urban residents in China, with CAD accounting for the predominant proportion (81.68%) (2). As reported by the American Heart Association (AHA), more than 20 million Americans suffer from CAD, among whom 805,000 estimated cases of myocardial infarction (MI), an acute manifestation of CAD, occur annually (3). Identifying severe CAD at an early stage is essential for reducing mortality. The precise and objective assessment of plaque burden in patients with CAD significantly affects prognosis. Given that current clinical practice for assessing CAD and its severity relies heavily on coronary computed tomography angiography (CCTA) or invasive coronary angiography (4), with limits on the feasibility of early and widespread risk stratification, there is a critical need for novel and non-invasive biomarkers to enable the identification of higher vascular plaque burden in CAD patients.

Insulin resistance (IR) is considered the major trait of type 2 diabetes mellitus (T2DM) (5). It promotes a systemic inflammatory state and endothelial dysfunction, thus leading to an atherogenic response (6–8). The triglyceride-glucose (TyG) index was proposed as a reliable biomarker for IR (9, 10). In previous studies (11, 12), the correlation between the TyG index and CAD was confirmed, but the TyG index was not associated with new-onset CAD severity, which was roughly assessed using the quantity of narrowed coronary arteries (12). The TyG index can be easily accessed from routine inpatient examinations at little cost, and it has the potential to identify CAD with high Gensini scores at early stages. The C-reactive protein-triglyceride-glucose index (CTI) is an index derived from the TyG index, and it is an innovative composite biomarker that integrates the levels of C-reactive protein (CRP) and the TyG index, thereby providing a combined estimate of both the systemic inflammatory status and the insulin resistance severity (13–16). The CTI was originally applied to predict survival in patients with malignant tumours (13). Regarding systemic arterial diseases, such as cardiovascular and cerebrovascular diseases, the CTI has been shown to significantly predict both the incidence and mortality of CAD globally (14–16). Furthermore, in the hypertensive population, the CTI is also a critical predictor of stroke (17). Another recent study demonstrated a significant correlation between the CTI and non-alcoholic fatty liver disease (NAFLD) (18). However, despite its ability to predict and estimate inflammation and metabolism-related diseases, the correlation between the CTI and the severity of CAD remains unexplored.

In addition, the atherogenic index of plasma (AIP), which is calculated from the triglyceride (TG) and high-density lipoprotein cholesterol (HDL-C) levels, is strongly associated with atherosclerotic burden and the development of cardiovascular disease (12, 19–21). The TyG index, CTI, and AIP are all associated with cardiovascular disease, and they share partially overlapping mechanisms, primarily involving insulin resistance, systemic inflammation, and atherogenic dyslipidaemia. However, no study to date has evaluated the combined effect of the TyG index, CTI, and AIP in the context of a quantitative assessment of coronary artery disease severity.

Linear and logistic regression approaches have been widely employed to investigate the association between metabolic parameters and binary clinical endpoints in previous studies (11, 12). However, both linear and logistic regression models have inherent limitations (22). Linear regression assumes a strictly linear relationship (23), which may not hold true in complex biological systems, whereas logistic regression relies on dichotomizing continuous outcomes (24), leading to a loss of granular information on disease severity. These results do not provide sufficient information about the quantitative association with CAD severity. Consequently, quantile regression, which offers greater flexibility in estimating exposure–outcome associations across the entire distribution of the outcome (25, 26), has been applied in medical research. Despite these advantages, no study has employed quantile regression to investigate the association between the TyG index and its derived indices and CAD severity.

On this basis, we propose the hypothesis that the combination of the TyG index, CTI, and AIP may provide a more comprehensive assessment of risk and that they all have quantile heterogeneity.

## 2 Methods

### 2.1 Study population and ethical statement

**Figure 1:**
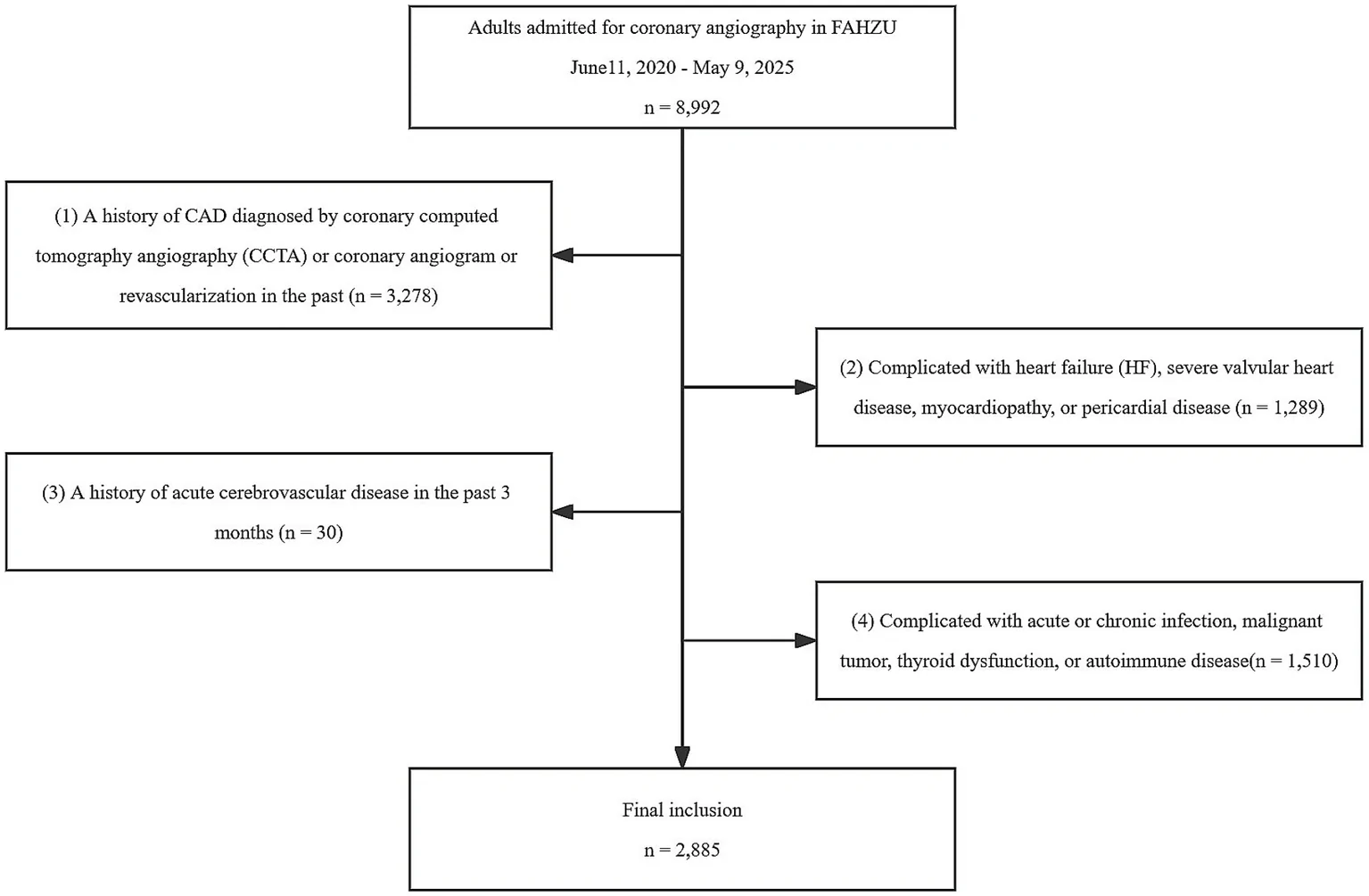
Inclusion and exclusion criteria. The Fourth Affiliated Hospital of Zhejiang University School of Medicine, FAHZU; Coronary artery disease, CAD; Coronary computed tomography angiography, CCTA; Heart failure, HF.

This study included 2,885 participants with newly diagnosed CAD, as confirmed by coronary angiography, from the Fourth Affiliated Hospital of Zhejiang University School of Medicine (FAHZU) between 11 June, 2020 and 9 May, 2025 (shown in Figure 1). The exclusion criteria included the following: (1) a history of CAD diagnosed by CCTA or coronary angiography or revascularization in the past (*n* = 3,278); (2) heart failure, severe valvular heart disease, myocardiopathy, or pericardial disease (*n* = 1,289); (3) a history of acute cerebrovascular disease in the past 3 months (*n* = 30); and (4) acute or chronic infection, malignant tumour, thyroid dysfunction, or autoimmune disease (*n* = 1,510). The protocol for this study adhered to the tenets of the Declaration of Helsinki. Formal approval was obtained from the Institutional Review Board (IRB) and Ethics Committee of FAHZU (Approval No. K2025263). Given its retrospective design, the need for informed consent was formally waived in accordance with pertinent national regulations and institutional stipulations. Patient data were retrieved from the hospital’s electronic medical records system, using the International Classification of Diseases, 10th Revision (ICD-10) for case identification. All laboratory examinations were conducted in the clinical laboratory of FAHZU.

### 2.2 Calculation of the TyG index and its derived indices

The TyG index is considered a measure of IR, and its calculation is as follows: Ln (TG [mg/dL] × fasting blood glucose (FBG) [mg/dL]/2) (9, 10). As its derived index, the CTI was used to reflect systemic inflammation and IR and was calculated as follows: 0.412 × Ln (CRP [mg/L]) + Ln (TG [mg/dL] × FBG [mg/dL]/2) (10, 14, 15). The AIP is another novel biomarker of atherosclerotic risk and is expressed by the following formula: Log_10_ (TG [mg/dL]/HDL-C [mg/dL]) (27). TyG-AIP is defined as the product of the TyG and normalized AIP. Similarly, the CTI-AIP is defined as the product of CTI and AIP. These products further support the synergistic interaction between inflammation-, glucose-, and lipid-related metabolic pathways.

AIP values may be either negative or positive, reflecting the relative balance between the TG and HDL-C levels. To standardize its scale and facilitate integration into the composite index, AIP was rescaled to a 0–1 interval using min–max normalization. This approach minimizes the potential distortion introduced by negative values and improves the comparability and interpretability of the combined metric, as previously reported by Chen X et al. (28).

All the calculation formulas are listed below (Equation 1).

$$\begin{array}{c} \mathrm{TyG}=\ln(\mathrm{TG}\times \mathrm{FBG}÷2)\\ \mathrm{CTI}=0.412\times \ln\mathrm{CRP}+\ln(\mathrm{TG}\times \mathrm{FBG}÷2)=0.412\times \ln\mathrm{CRP}+\mathrm{TyG}\\ \mathrm{AIP}=\log_{10}\mathrm{TG}/(\mathrm{HDL}-C)\;\\ \mathrm{TyG}-\mathrm{AIP}=\mathrm{TyG}\times \mathrm{AIP}\\ \mathrm{CTI}-\mathrm{AIP}=\mathrm{CTI}\times \mathrm{AIP} \end{array}$$ (1)

Equation 1: Calculation of TyG and its derived indices. The “-” in HDL-C, TyG-AIP and CTI-AIP is not used as an operation symbol.

### 2.3 Calculation of Gensini score

As modified by Rampidis GP et al. in 2019, the Gensini score was first described in 1975 as a means of quantifying the severity of CAD based on angiography images (29, 30). In general, 3 dimensions are needed to assess coronary plaque burden: the severity of obstruction, multiple regional factors and multiple collateral adjustment factors (29). The detailed calculation for the Gensini score is listed in Supplementary material 1 Gensini Score.

### 2.4 Covariates

Medical experts systematically collected baseline data and recorded them in the electronic medical records system as previously described (31), including (1) demographic and lifestyle characteristics such as sex, age, smoking status, and alcohol use and (2) body measurements such as height (centimetres/cm) and weight (kilograms/kg). Body mass index (BMI) was calculated as follows (Equation 2): (3) Clinical history: Documented hypertension, diabetes and stroke diagnoses, along with current medications.

$$\mathrm{BMI}=\frac{\text{Weight}}{\text{Heigh}t^{2}}$$ (2)

Equation 2: The calculation of BMI. Height is measured in metres. Weight is measured in kilograms.

### 2.5 Data preprocessing and multiple imputation

Prior to statistical analysis, data preprocessing was performed to address missing values and measurement limit issues. Categorical variables (sex, hypertension, diabetes, stroke, smoking history, alcohol history, blood pressure management, and lipid medication use) were converted to factor type for appropriate analysis. Clinical assay results below the detection limit were recoded to ensure valid numerical analysis. Given the analytical equivalence, measurements of hypersensitive C-reactive protein (hs-CRP) and conventional CRP were merged into a single variable (hs-CRP/CRP) using the available quantitative results (32). Missing data were handled using multiple imputation (33). Candidate variables for imputation included demographic characteristics, clinical history, laboratory parameters, and anthropometric measures (e.g., leukocyte count, lipid profiles, renal function markers, glycated haemoglobin (HbA1c), and body mass index (BMI)). Missing data patterns were initially examined, followed by multiple imputation using predictive mean matching (PMM) implemented in the mice package. Missing rates were reported in Supplementary Data sheet 2. Five imputed datasets were generated with 10 iterations each. Statistical analyses were performed independently in each imputed dataset, and regression coefficients, standard errors, and confidence intervals were pooled according to Rubin’s rules. For heterogeneity tests across quantiles, Wald statistics from each imputed dataset were combined using the D2 pooling method to obtain pooled test statistics and *p* values. Imputation quality was verified by comparing missing value counts before and after the procedure, confirming successful handling of all missing entries. The process was performed using R software (version 4.4.3; R Foundation for Statistical Computing).

### 2.6 Statistical analysis

Continuous variables with normal and non-normal distributions are expressed as the means ± standard deviation and median (interquartile range), respectively. Categorical variables are summarized as numbers (percentages).

Associations between the severity of coronary atherosclerosis, as quantified by the Gensini score, and key metabolic indices (the TyG index and its derived composite indices) were evaluated using correlation analysis. Pairwise Pearson correlation coefficients were calculated using complete observations (use = “complete.obs”) to assess the strength and direction of the linear relationships. The resulting correlation matrix was melted into a long format and visualized as a heat map using the ‘ggplot2’ package. All the statistical tests were two-tailed, and a *p* value <0.05 was considered to indicate statistical significance.

Owing to the skewed distribution of the Gensini score, quantile regression was employed to investigate the associations between metabolic indices and coronary atherosclerosis severity across the entire distribution of the outcome. Quantile regression models were fitted at the 10th through 90th percentiles (*τ* = 0.10, 0.20, 0.30, 0.40, 0.50, 0.60, 0.70, 0.80, and 0.90) of the Gensini score distribution to examine the association of the TyG index and its derived indices with CAD severity. Quantile regression is a flexible and powerful tool that estimates how CAD relates to the TyG index and its derived indices at any quantile of the Gensini score distribution, that is, the Gensini score at the lower end, midrange, higher end, or anywhere within its conditional distribution (22, 34). Coefficient estimates with 95% confidence intervals were derived for each quantile. To illustrate the heterogeneous effects visually, conditional quantile plots were generated using the ‘ggplot2’ package, showing the relationships between selected indices (e.g., CTI and TyG) and the Gensini score across the specified quantiles. Statistical significance was defined as a two-sided *p* < 0.05. All analyses were performed using R software with the ‘quantreg’ package.

To account for potential confounding factors, multivariable quantile regression analyses were performed to evaluate the associations between each metabolic index and the Gensini score across the 10th–90th quantiles (*τ* = 0.10–0.90). Separate models were fitted for each metabolic index to avoid multicollinearity arising from the high correlations among composite indices. All the models were adjusted for age, sex, body mass index (BMI), hypertension status, diabetes mellitus status, smoking status, antihypertensive medication use, lipid-lowering medication use, and antidiabetic medication use. Regression coefficients (*β*) with corresponding 95% confidence intervals were estimated at each quantile.

To assess heterogeneity formally in the associations across the conditional distribution of the Gensini score, analysis of variance for quantile regression (anova.rq) was conducted after fitting the quantile regression models. The null hypothesis was that the regression coefficients were equal across all the quantiles examined here (*τ* = 0.10–0.90). A two-sided *p* < 0.05 indicated significant heterogeneity of regression slopes across quantiles.

All analyses were two-tailed, and a *p*-value <0.05 was considered statistically significant. These analyses were performed using R software (version 4.4.3; R Foundation for Statistical Computing).

## 3 Results

### 3.1 Baseline characteristics

A total of 2,885 patients who underwent coronary angiography were included and stratified into quartiles according to the TyG index (Q1–Q4, *n* ≈ 721 per group). As shown in Table 1, the patients in the higher TyG quartiles were progressively younger and had a higher BMI (both *p* < 0.001). The proportion of male patients increased modestly across the TyG index quartiles (*p* = 0.016). The incidence of hypertension and diabetes mellitus increased significantly with increasing levels of the TyG index (*p* = 0.002 and *p* < 0.001, respectively), with diabetes showing a marked gradient from Q1 to Q4. A history of stroke differed modestly across quartiles (*p* = 0.001), whereas alcohol consumption did not vary significantly among groups (*p* = 0.496). Current smoking was more prevalent in higher TyG quartiles, while the proportion of never smokers decreased (*p* < 0.001). The use of antihypertensive therapy showed a borderline difference across quartiles (*p* = 0.071), whereas the use of lipid-lowering medication was comparable among the groups (*p* = 0.175). In contrast, the proportion of patients who received antidiabetic therapy increased substantially in the higher TyG quartiles (*p* < 0.001). Overall, patients with a high TyG index exhibited a less favourable cardiometabolic risk profile, as characterized by higher adiposity, a greater burden of diabetes and hypertension, and a higher prevalence of active smoking.

**Table 1:** Baselines of the included patients.

Characteristics	Quantiles of TyG					
	Total number	Q1	Q2	Q3	Q4	*p* value
*n*	2,885	722	721	721	721	
Age [mean (SD)]	61.14 (10.99)	63.29 (11.14)	62.16 (10.79)	60.22 (10.61)	58.88 (10.90)	<0.001
BMI [mean (SD)]	25.14 (3.49)	23.83 (3.48)	24.93 (3.64)	25.57 (3.04)	26.24 (3.33)	<0.001
Sex, %						0.016
Male	1,677 (58.1)	431 (59.7)	385 (53.4)	419 (58.1)	442 (61.3)	
Female	1,208 (41.9)	291 (40.3)	336 (46.6)	302 (41.9)	279 (38.7)	
Hypertension (%)						0.002
Non-hypertensive	1,236 (42.8)	345 (47.8)	321 (44.5)	290 (40.2)	280 (38.8)	
Hypertensive	1,649 (57.2)	377 (52.2)	400 (55.5)	431 (59.8)	441 (61.2)	
Diabetes (%)						<0.001
Non-diabetic	2,326 (80.6)	648 (89.8)	605 (83.9)	590 (81.8)	483 (67.0)	
Diabetic	559 (19.4)	74 (10.2)	116 (16.1)	131 (18.2)	238 (33.0)	
Stroke (%)						0.001
No history of CVD	2,711 (94.0)	657 (91.0)	680 (94.3)	687 (95.3)	687 (95.3)	
Stroke survivor	174 (6.0)	65 (9.0)	41 (5.7)	34 (4.7)	34 (4.7)	
History of smoking (%)						<0.001
Non-smoker	1942 (67.3)	507 (70.2)	508 (70.5)	475 (65.9)	452 (62.7)	
Current smoker	605 (21.0)	128 (17.7)	117 (16.2)	165 (22.9)	195 (27.0)	
Ex-smoker	338 (11.7)	87 (12.0)	96 (13.3)	81 (11.2)	74 (10.3)	
History of alcohol (%)						0.496
Never drinker	2015 (69.8)	520 (72.0)	507 (70.3)	490 (68.0)	498 (69.1)	
Current drinker	734 (25.4)	165 (22.9)	181 (25.1)	194 (26.9)	194 (26.9)	
Ex-drinker	136 (4.7)	37 (5.1)	33 (4.6)	37 (5.1)	29 (4.0)	
BPM (%)						0.071
Treatment-Naïve	1,381 (47.9)	375 (51.9)	344 (47.7)	330 (45.8)	332 (46.0)	
On therapy	1,504 (52.1)	347 (48.1)	377 (52.3)	391 (54.2)	389 (54.0)	
Lipid medication (%)						0.175
Treatment-Naïve	1881 (65.2)	453 (62.7)	462 (64.1)	476 (66.0)	490 (68.0)	
On therapy	1,004 (34.8)	269 (37.3)	259 (35.9)	245 (34.0)	231 (32.0)	
Diabetic medication (%)						<0.001
Treatment-Naïve	2,391 (82.9)	648 (89.8)	617 (85.6)	606 (84.0)	520 (72.1)	
On therapy	494 (17.1)	74 (10.2)	104 (14.4)	115 (16.0)	201 (27.9)	

Body mass index, BMI; Cerebrovascular disease, CVD; Medication for blood pressure, BPM. Diabetic medications include insulin injection.

### 3.2 Correlation analysis

**Figure 2:**
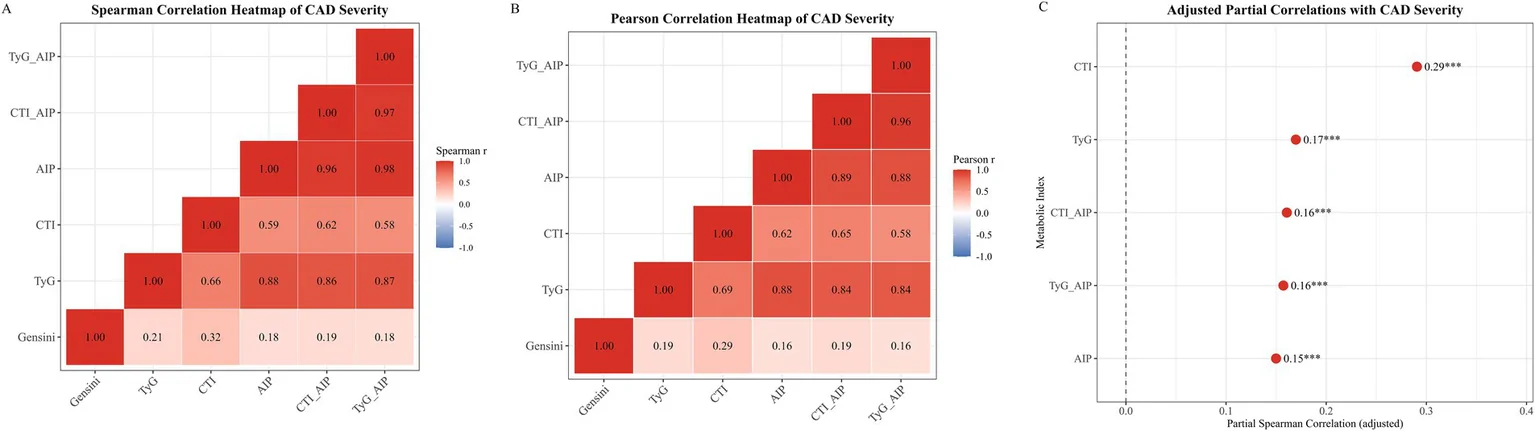
Correlation heat map of TyG, its derived indices and atherosclerosis severity (assessed by Gensini score). **(A)** Spearman correlation analysis; **(B)** Pearson correlation analysis; **(C)** partial adjusted correlation analyses, adjusted for hypertension, diabetes, stroke, smoking history, and BMI. *** Means *p* < 0.001.

Pearson correlation analysis demonstrated that the Gensini score was positively correlated with the TyG index, CTI, AIP, and their derived composite indices (Figure 2B). The TyG index demonstrated a positive and weak linear correlation with the Gensini score. The CTI showed a positive and more linear association with the Gensini score (*r* ≈ 0.29), suggesting a stronger link between inflammatory–metabolic dysregulation and angiographic disease severity. The AIP was similarly correlated with the Gensini score as the TyG index was, reflecting the contribution of atherogenic lipid profiles to the coronary lesion burden. Furthermore, composite indices combining the TyG index or CTI with the AIP remained positively associated with the Gensini score, underscoring the cumulative relationship between metabolic disturbances and the extent of coronary atherosclerosis.

Given the skewed distribution of the Gensini score, a Spearman correlation analysis was performed (Figure 2A). Compared with the TyG alone, the composite index TyG-AIP demonstrated a modest increase in correlation strength (partial *r* ≈ 0.22 vs. 0.19), suggesting that incorporating lipid-related information slightly enhances the association with the Gensini score.

To minimize the influence of confounding factors, partial correlation analyses (Figure 2C) adjusted for hypertension, diabetes, stroke, smoking history, and BMI consistently demonstrated significant associations between metabolic indices and the Gensini score. The CTI exhibited the strongest correlation with the Gensini score among all the evaluated indices (partial *r* ≈ 0.29), while the composite index CTI-AIP showed a moderate decrease compared with the CTI alone (partial *r* ≈ 0.16 vs. 0.29). Across all panels, the TyG and its derived or composite indices remained positively correlated with coronary atherosclerosis severity after adjustment (all ***, *p* < 0.001).

### 3.3 Quantile regression analysis

**Figure 3:**
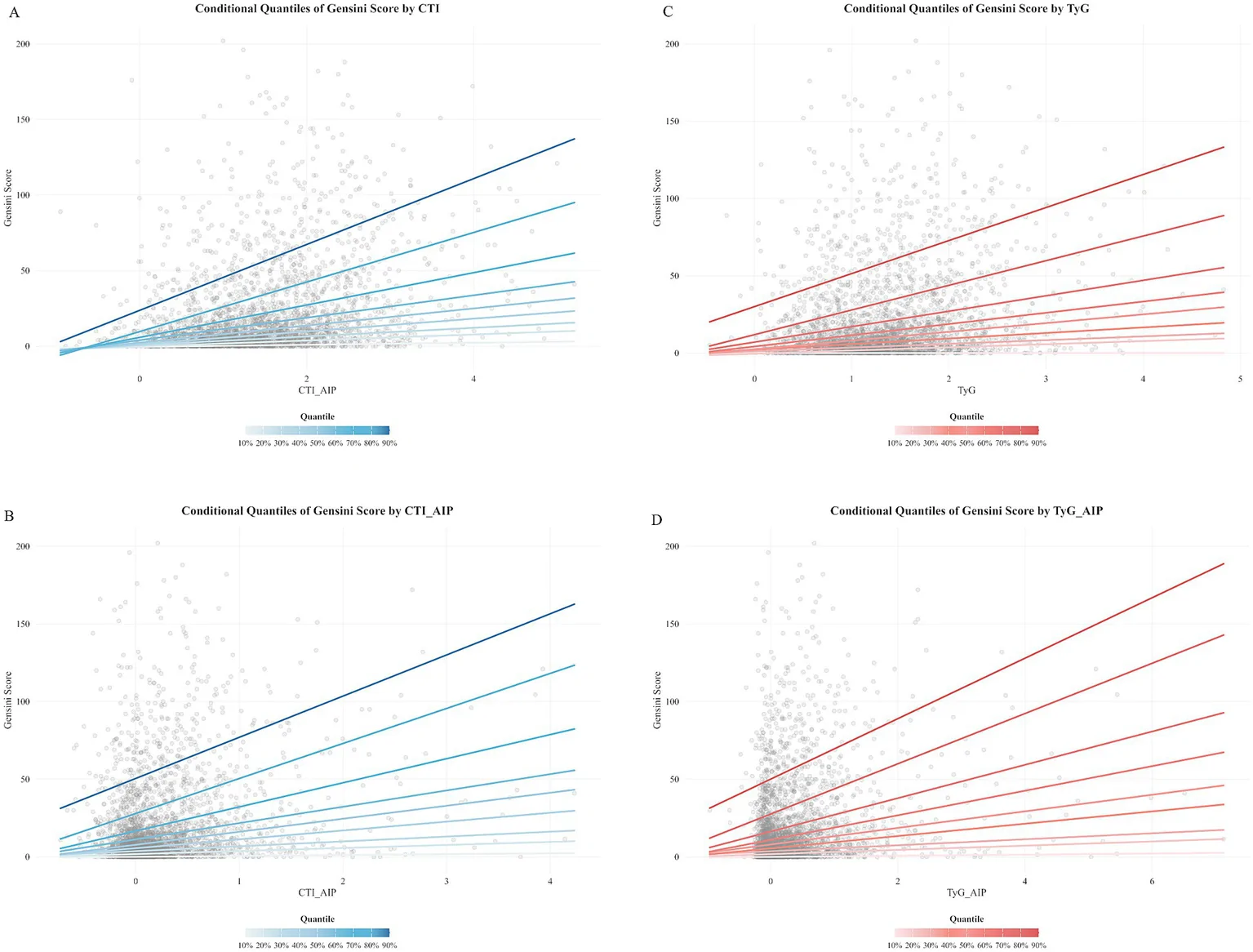
Conditional quantiles of the TyG index (C) and its derived indices combined with AIP **(A–D)**.

Building upon the modest linear correlations observed between metabolic indices and the Gensini score (Figure 3), quantile regression analyses were performed to characterize their associations across the conditional distribution of angiographic atherosclerosis severity. As shown in Figure 3, quantile regression analyses revealed marked heterogeneity in the associations between metabolic indices and coronary atherosclerotic burden across the distribution of Gensini scores. For all indices examined here, the regression slopes progressively increased from lower to higher conditional quantiles of the Gensini score. Among the individual indices, the CTI demonstrated consistently steeper slopes than the TyG index demonstrated across most quantiles (Figures 3A,C), particularly in the upper tail of the Gensini distribution, suggesting a stronger and more sensitive relationship with advanced atherosclerotic burden. This gradient became especially pronounced at higher quantiles, in which incremental increases in CTI were associated with disproportionately larger increases in the Gensini score. Notably, the composite indices further accentuated this pattern. Compared with their corresponding single indices, both CTI-AIP and TyG-AIP exhibited substantially steeper slopes (Figures 3B,D), with the greatest effect observed for CTI-AIP. Similarly, TyG-AIP (Figure 3D) outperformed TyG alone, particularly in the upper quantiles, underscoring the added value of integrating lipid-related information into glucose-triglyceride-based metrics. Collectively, these findings demonstrate a clear dose–response and risk–amplification pattern, whereby the associations between metabolic indices and atherosclerotic burden increase progressively with increasing disease severity. Composite indices, especially CTI-AIP, may provide enhanced clinical utility for identifying patients at highest risk.

**Figure 4:**
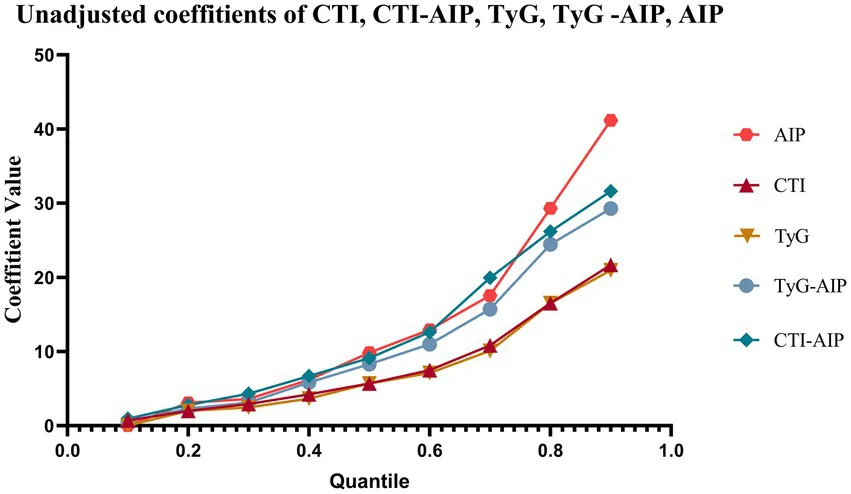
Unadjusted quantile regression coefficients of the AIP, CTI, TyG, CTI-AIP, and TyG-AIP.

To characterize the associations between metabolic indices and the conditional distribution of the Gensini score, unadjusted quantile regression analyses were performed across the 10th–90th quantiles. As shown in Figure 4, all the indices exhibited progressively larger regression coefficients with increasing quantiles of the Gensini score, indicating that their associations varied across the outcome distribution rather than remaining constant.

Among the individual indices, TyG demonstrated a marked increase in regression coefficients from the lower to upper quantiles, suggesting a progressively stronger association at higher conditional quantiles of the Gensini score. The CTI showed a similar but less pronounced trend, with relatively smaller coefficient estimates throughout the quantile range. The incorporation of AIP further enhanced these associations, as both TyG-AIP and CTI-AIP displayed steeper coefficient trajectories than their corresponding single indices. In particular, CTI-AIP exhibited the largest coefficient estimates across most quantiles, whereas TyG-AIP also showed a substantial increase towards the upper quantiles, indicating that integrating lipid-related information strengthened the relationship with the Gensini score.

Overall, the unadjusted analyses clearly revealed heterogeneity in the associations between metabolic indices and the Gensini score across conditional quantiles, suggesting that the magnitude of the association differed substantially along the distribution of the coronary atherosclerotic burden.

### 3.4 Multivariable-adjusted quantile regression analysis

To determine whether these heterogeneous associations were independent of conventional cardiovascular risk factors, multivariable quantile regression models were further adjusted for age, sex, BMI, hypertension, diabetes, smoking history, antihypertensive medication, lipid-lowering medication, and antidiabetic medication.

**Figure 5:**
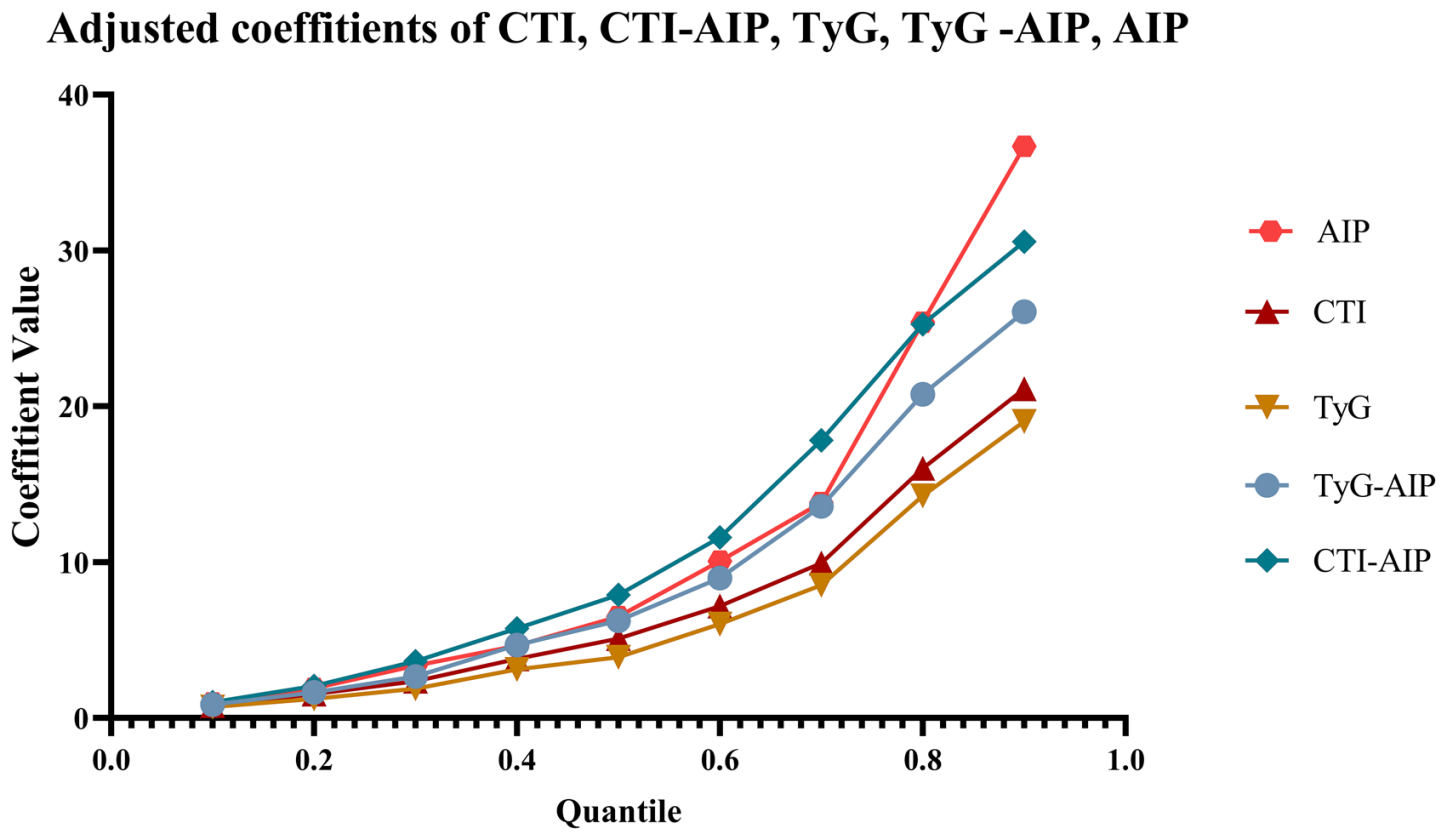
Quantile regression coefficients of the AIP, CTI, TyG, CTI-AIP, and TyG-AIP. They were adjusted for multiple covariates, including age, sex, BMI, hypertension, diabetes, smoking history, antihypertension medication, lipid-lowering medication, and antidiabetic medication.

As shown in Figure 5, the positive associations observed in the unadjusted analyses remained statistically significant after multivariable adjustment across nearly all the conditional quantiles. Although the regression coefficients were attenuated compared with those of the crude models, all the indices continued to demonstrate progressively increasing effect sizes from lower to higher quantiles.

Among the individual indices, the TyG remained positively associated with the Gensini score across all quantiles, with *β* increasing from 0.700 (95% CI, 0.258–1.143) at *τ* = 0.10 to 19.035 (95% CI, 13.591–24.478) at *τ* = 0.90 (all *p* < 0.01). The CTI showed a similar but consistently stronger association than the TyG after adjustment, with *β* increasing from 0.796 (95% CI, 0.493–1.099) to 21.117 (95% CI, 16.468–25.132) across the same quantiles. The composite indices demonstrated the greatest effect sizes after adjustment. TyG-AIP exhibited increasing regression coefficients from 0.849 (95% CI, 0.179–1.518) at *τ* = 0.10 to 26.063 (95% CI, 19.914–32.212) at *τ* = 0.90, whereas CTI-AIP consistently showed the strongest association among all the evaluated indices, with coefficients rising from 1.017 (95% CI, 0.395–1.639) to 30.561 (95% CI, 24.271–36.851) across the quantile spectrum (all *p* < 0.001). These findings indicate that combining inflammatory and lipid-related information substantially strengthened the association with the Gensini score compared with that with either individual index alone.

Compared with the unadjusted models, multivariable adjustment did not diminish the progressive increase in regression coefficients across conditional quantiles. Instead, the increasing trajectories became more distinct after controlling for demographic characteristics, cardiometabolic risk factors, and medication use. Notably, the AIP exhibited the largest regression coefficient at the highest conditional quantile (*β* = 36.697, 95% CI 24.064–49.329), whereas the CTI-AIP showed consistently large effect estimates throughout the quantile spectrum. In addition, both composite indices (CTI-AIP and TyG-AIP) maintained the steepest increases towards the upper quantiles, indicating that their stronger associations were robust and independent of the measured confounding factors.

### 3.5 Heterogeneity across quantiles

Analysis of the testing slope equality across quantiles (anova.rq) revealed significant heterogeneity in regression coefficients across quantiles for all metabolic indices (all *p* values for heterogeneity <0.001), indicating that the associations with the Gensini score varied significantly according to the atherosclerosis burden (Table 2). Among the evaluated indices, CTI showed the greatest heterogeneity (*f* = 9.98), followed by TyG (*f* = 6.16), CTI-AIP (*f* = 5.49), and TyG-AIP (*f* = 5.13). These findings indicate that the effects of metabolic dysfunction on coronary atherosclerotic burden were not constant across the distribution of the Gensini score and became progressively stronger in patients with higher conditional quantiles.

**Table 2:** Heterogeneity across quantiles.

Index	*f* value	*p* value	Significance
AIP	3.02135895771966	0.0249	*
TyG	6.163590051	<0.001	***
CTI	9.98228453895079	<0.001	***
TyG-AIP	5.13273321732099	<0.001	***
CTI-AIP	5.49572777530061	<0.001	***

*p < 0.05. Unadjusted heterogeneity is provided in Supplementary material 3.

## 4 Discussion

This study is the first large-scale investigation to reveal a significant positive association of metabolic indices, including the TyG index and its derived indices, with the severity of CAD being assessed by angiography, demonstrating quantile regression results from an additional perspective. Moreover, to our knowledge, this is the first study employing quantile regression to explore the quantile heterogeneity in the associations of four indices with CAD severity in newly diagnosed patients. Several important findings emerged from our analysis. Overall, all the evaluated metabolic indices showed a weak positive linear association with the Gensini score, which indicated that worsening metabolic dysfunction, such as IR and systemic inflammation, was associated with increased coronary atherosclerotic burden. Quantile regression analyses revealed significant heterogeneity in the associations between metabolic indices and coronary atherosclerotic burden across the distribution of Gensini scores. For all indices examined herein, regression slopes increased progressively from lower to higher conditional quantiles, indicating that the strength of association was substantially amplified among patients with higher Gensini scores. Among the evaluated indices, all metabolic markers demonstrated progressively increasing regression coefficients across the conditional distribution of the Gensini score in the unadjusted quantile regression analyses, indicating stronger associations at higher quantiles. At the 90th quantile, the regression coefficients reached 18.95 for the TyG index, 20.49 for CTI, 26.79 for TyG-AIP, and 31.66 for CTI-AIP, indicating that the composite indices consistently exhibited stronger associations than their corresponding individual indices. Compared with the individual indices, the incorporation of AIP further strengthened these associations, as both TyG-AIP and CTI-AIP exhibited consistently larger regression coefficients than their corresponding single indices across most quantiles. In particular, CTI-AIP showed the largest coefficient estimates throughout the upper quantiles, followed by TyG-AIP, whereas CTI also demonstrated stronger associations than TyG did at higher quantiles. These findings suggest that integrating lipid-related information enhanced the association of both TyG- and CTI-based indices with the conditional distribution of the Gensini score.

Multivariable-adjusted quantile regression analyses further strengthened our findings by demonstrating that the associations between metabolic indices and the Gensini score remained significant after adjusting for conventional cardiovascular risk factors and medication use. Although the regression coefficients were attenuated compared with those of the unadjusted models, the overall quantile-dependent patterns were preserved, suggesting that these associations were not solely explained by demographic characteristics, metabolic comorbidities, or pharmacological treatment. Notably, the regression coefficients of the composite indices (CTI-AIP and TyG-AIP) consistently exhibited larger regression coefficients than their corresponding individual indices across the conditional distribution of the Gensini score, indicating that integrating lipid-related information into inflammation- and insulin resistance-based markers provides incremental value for characterizing coronary atherosclerotic burden. Furthermore, the significant heterogeneity identified by the quantile regression analysis of variance indicates that the effects of these metabolic indices are not constant across the conditional distribution of the Gensini score. Rather than assuming a single average effect, quantile regression revealed that the strength of association varied across different conditional quantiles, highlighting the importance of evaluating distribution-specific effects when the relationship between metabolic dysfunction and coronary atherosclerosis is being assessed.

Taken together, among the evaluated indices, CTI-AIP demonstrated the strongest and most consistent associations across most quantiles, suggesting that simultaneously capturing systemic inflammation, insulin resistance, and atherogenic dyslipidaemia may provide additional comparative insight beyond individual metabolic markers. These exploratory findings warrant confirmation in future studies with formal discrimination testing.

The TyG index, a widely adopted biomarker of IR due to its favourable combination of computational simplicity, sensitivity, and diagnostic specificity, was consistent with the associations with risks such as cardiovascular disease mortality (35–37) and the development and progression of atherosclerotic cardiovascular disease (12) reported in previous studies. IR, the major characteristic of T2DM, plays a vital role in the pathophysiology of atherosclerosis (12, 38, 39). A possible explanation is that IR promotes endothelial dysfunction (40, 41) by over-activating oxidative stress and platelet hyperactivity (42). These processes facilitate lipid deposition within the arterial wall and accelerate plaque formation and progression (41, 43). In previous studies, whether the TyG index is significantly correlated with CAD severity has been controversial (12). The present study, which precisely assessed CAD severity, extends these observations by demonstrating that the relationship between the TyG index and coronary atherosclerosis is not uniform across disease severity and becomes substantially stronger among individuals with more advanced lesions.

In addition, in taking inflammatory–metabolic imbalance into consideration, CTI was incorporated into our analysis. As a central process in disease-specific mechanisms, chronic systemic inflammatory activation promotes lipid accumulation, endothelial injury, and plaque instability (44, 45) via the release of immune cell cytokines (46). The robust association between CTI and CAD severity observed in our study reinforces the concept that chronic systemic inflammation acts as a critical determinant of atherosclerotic burden, extending beyond traditional lipid-centric pathways. Dyslipidaemia is another key contributor to the development of atherosclerosis (47). Positive associations between dyslipidaemia indices, including AIP and atherosclerosis were also well documented in previous research (48–50). AIP is calculated from TG and HDL-C levels, mirroring the equilibrium between plasma atherogenic and antiatherogenic factors (19, 51, 52) and providing an index of the level of small dense low-density lipoprotein (sdLDL) particles (53). These findings further confirm the association between lipid metabolism disorders and the risk of cardiovascular disease. In our study, the positive association between the AIP and the Gensini score supports the role of dyslipidaemia in promoting the coronary atherosclerotic burden. The observed positive correlations between these indices and the Gensini score in our study further highlight the concept that metabolic and inflammatory disturbances jointly contribute to coronary atherosclerotic progression. While previous studies have typically evaluated single biomarkers or metabolic indices, the combination of the TyG index or CTI with the AIP in the present analysis, such as CTI-AIP, reveals that integrating multiple metabolic pathways may improve the assessment of atherosclerotic burden. Furthermore, the progressive increase in regression slopes across higher Gensini quantiles suggests that these metabolic disturbances may exert a more pronounced effect during the advanced stages of CAD, when cumulative vascular injury and plaque burden are already substantial.

From a clinical perspective, identifying patients with CAD with high Gensini scores remains a critical and persistent challenge. Consequently, for the first time, 2,885 patients whose CAD was confirmed by coronary angiography were selected for this study. The utility of the TyG index as a simple and accessible biomarker for identifying CAD patients with high Gensini scores was verified in this study, and its quantile heterogeneity improved the exploratory identification of potential risk markers in CAD patients. Moreover, unlike single biomarkers that reflect only isolated aspects of metabolism or inflammation, composite indices, such as TyG-AIP and CTI-AIP, which simultaneously capture the synergistic interplay among IR, chronic systemic inflammation, and atherogenic dyslipidaemia, show potential advantages in CAD. By encapsulating multiple pathophysiological pathways within a single measure, they provide a holistic assessment of the atherosclerotic burden. Thus, these biomarkers may be particularly useful for identifying individuals with multiple metabolic risk factors, providing additional information for personalized risk assessment and guiding clinical decision-making.

## 5 Strengths and limitations

This study has several strengths. First, the inclusion of patients with newly diagnosed CAD minimized the potential confounding effect of secondary prevention medications, thereby allowing for a more accurate assessment of native biomarker levels. On this basis, the analysis was conducted on a relatively large sample, enhancing the statistical power and generalizability of the findings. Second, by employing the Gensini score derived from coronary angiography, we were able to provide a more objective and quantitative assessment of atherosclerotic severity than studies relying on binary clinical endpoints. Third, to our knowledge, this is the first study employing quantile regression to explore the quantile heterogeneity in these associations across the full spectrum of CAD severity, revealing patterns that conventional regression models might have overlooked. Lastly, the simultaneous evaluation of both single and composite metabolic indices provided a broader understanding of the relationships between cardiometabolic dysfunction and coronary atherosclerosis.

Despite the strengths above, several limitations should also be acknowledged. First, the cross-sectional design of the study precludes the establishment of causal relationships between metabolic indices and coronary atherosclerosis. Second, the study population consisted of patients who underwent coronary angiography in a single centre in eastern China, with the majority presenting with clinical symptoms, which may limit the generalizability of the findings to the global population or asymptomatic individuals. Third, although multiple clinical variables were considered, the possibility of residual confounding from unmeasured factors, such as diet, medication, and lifestyle, cannot be entirely excluded. Fourth, although multiple imputation was performed and regression estimates were pooled using Rubin’s rules, there is currently no universally accepted framework for pooling quantile regression estimates after multiple imputation. Therefore, although our approach follows current methodological recommendations, the pooled quantile regression results should be interpreted with appropriate caution. This approach may lead to underestimated standard errors and increased uncertainty in the estimates, as it fails to account for between-imputation variability. Fifth, the Min-Max scaling applied to AIP is a sample-specific linear transformation, and therefore our findings should be interpreted as reflecting relative risk rankings within the present cohort rather than providing externally applicable absolute cut-offs. Moreover, as a retrospective observational study, the present study was unable to establish causality. Lastly, the absence of longitudinal follow-up data prevented us from evaluating the predictive value of these metabolic indices for future cardiovascular events.

## 6 Conclusion and future prospects

In conclusion, this study demonstrated that metabolic indices reflecting insulin resistance, inflammation, and dyslipidaemia, including the TyG index, CTI, and AIP, are significantly and positively associated with the severity of CAD in patients with newly diagnosed CAD. Importantly, the use of quantile regression demonstrated that the strength of these associations increases progressively in higher Gensini score quantiles. The TyG index has the strongest effect in patients with moderate to relatively high Gensini scores. TyG-AIP demonstrated a nonlinear, progressively strengthening association with the Gensini score, particularly when it was marked beyond the 70th percentile. Collectively, these findings support the clinical value of simple and accessible metabolic biomarkers, particularly combined indices, for providing additional comparative insight beyond individual metabolic markers. Future prospective and multicentre studies are warranted to confirm these findings and to evaluate their ability to predict long-term cardiovascular outcomes.

## Data Availability

The datasets presented in this article are not readily available because privacy or ethical restrictions. Requests to access the datasets should be directed to Yunpeng Jin, 8013013@zju.edu.cn.

## Glossary

AHA: American Heart Association
AIP: Atherogenic index of plasma
BMI: Body mass index
BPM: Medication for blood pressure
CAD: Coronary artery disease
CCTA: Coronary computed tomography angiography
CHD: Coronary heart disease
cm: Centimetre
COVID-19: Coronavirus disease 2019
CRP: C-reactive protein
CTI: The C-reactive protein-triglyceride-glucose index
CVD: Cerebrovascular disease
dL: Decilitre
FAHZU: The Fourth Affiliated Hospital of Zhejiang University School of Medicine
FBG: Fasting blood glucose
HbA1c: Glycated haemoglobin
HDL-C: High-density lipoprotein cholesterol
hs-CRP: Hypersensitive C-reactive protein
ICD-10: The International Classification of Diseases, 10th Revision
IR: Insulin resistance
IRB: Institutional Review Board
kg: Kilogram
mg: Milligram
MI: Myocardial infarction
NAFLD: Non-alcoholic fatty liver disease
PMM: Predictive mean matching
sdLDL: Small, dense low-density lipoproteins
SE: Standard Error
T2DM: Type 2 diabetes mellitus
TG: Triglyceride
TyG: Triglyceride-glucose
*τ*: Quantile
